# The C_i_C_s_(Si_I_)_n_ Defect in Silicon from a Density Functional Theory Perspective

**DOI:** 10.3390/ma11040612

**Published:** 2018-04-16

**Authors:** Stavros-Richard G. Christopoulos, Efstratia N. Sgourou, Ruslan V. Vovk, Alexander Chroneos, Charalampos A. Londos

**Affiliations:** 1 Faculty of Engineering, Environment and Computing, Coventry University, Priory Street, Coventry CV1 5FB, UK; ac0966@coventry.ac.uk; 2 Departmant of Physics, Section of Solid State Physics, National and Kapodistrian University of Athens, Panepistimiopolis Zografos, 157 84 Athens, Greece; e_sgourou@hotmail.com (E.N.S.); hlontos@phys.uoa.gr (C.A.L.); 3 V. N. Karazin Kharkiv National University, 4 Svobody sq., 61077 Kharkiv, Ukraine; rvvovk2017@gmail.com; 4 Department of Materials, Imperial College London, London SW7 2AZ, UK

**Keywords:** silicon, carbon, defects, density functional theory

## Abstract

Carbon constitutes a significant defect in silicon (Si) as it can interact with intrinsic point defects and affect the operation of devices. In heavily irradiated Si containing carbon the initially produced carbon interstitial–carbon substitutional (C_i_C_s_) defect can associate with self-interstitials (Si_I_’s) to form, in the course of irradiation, the C_i_C_s_(Si_I_) defect and further form larger complexes namely, C_i_C_s_(Si_I_)_n_ defects, by the sequential trapping of self-interstitials defects. In the present study, we use density functional theory to clarify the structure and energetics of the C_i_C_s_(Si_I_)_n_ defects. We report that the lowest energy C_i_C_s_(Si_I_) and C_i_C_s_(Si_I_)_2_ defects are strongly bound with −2.77 and −5.30 eV, respectively.

## 1. Introduction

For more than six decades, the rapid evolution of microelectronics has constituted Si as a prevalent material. In the past few years, however, the aim has been to replace Si with other substrates (such as germanium) in nanoelectronic applications. Nevertheless, Si will remain the mainstream material for sensors and photovoltaics [1,2,3,4,5,6,7,8,9,10]. From a fundamental solid-state physics perspective, the study of point defects and defect clusters in Si remains of interest, as these can impact its materials properties. This is also technologically motivated, as in order to optimise devices, it is necessary to control the oxygen-related defects (vacancy-oxygen interstitial pairs, VO) as well as the carbon-related defects (such as C_i_C_s_(Si_I_)_n_ and C_i_O_i_(Si_I_)_n_, n = 1, 2, …) [11,12,13,14,15,16,17,18,19,20,21,22,23,24,25]. 

It is established that carbon in Si has a strong tendency to associate with self-interstitials (Si_I_’s). Indeed, carbon-related defects produced by the irradiation, such as C_i_O_i_, C_i_C_s_, and C_i_, readily trap Si_I_’s leading to the formation of larger complexes (for example C_i_O_i_(Si_I_)_n_, C_i_C_s_(Si_I_)_n_, and C_i_(Si_I_)_n_) [12,14]. This is important, as the existence of these defects restrains the formation of larger Si_I_ clusters, such as the (113) extended defects, which have detrimental effects in microelectronic devices [26]. Additionally, reaction processes involving the formation of C_i_O_i_(Si_I_)_n_, C_i_C_s_(Si_I_)_n_, and C_i_(Si_I_)_n_ structures are taken into account when radiation damage data are modeled for the fabrication of Si-tolerant radiation detectors [27,28].

The structure of the C_i_C_s_(Si_I_)_n_ complexes with n = 2, 3, … is not established, and the electrical activity of the members of the family is not known. The C_i_C_s_ center has been detected and reported by many experimental techniques [28]. Regarding the optical activity, at least 11 local vibration modes have been correlated with the different configurations of the defect, which exhibit metastability [28]. However, only two IR bands have been correlated with the C_i_C_s_(Si_I_) complex and another two with the C_i_C_s_(Si_I_)_2_ complex [16]. Regarding the electrical activity, at least two levels have been correlated with the C_i_C_s_ defect [28]. However, no levels have been associated with the C_i_C_s_(Si_I_)_n_ complexes, although it is not unreasonable to expect that levels will be introduced in the gap. To the best of our knowledge, there is no experimental or theoretical study proving that Si_I_’s passivate the C_i_C_s_ defect. Finally, the C_i_C_s_ defect exhibits metastability [28], although this behavior has not been detected for the C_i_C_s_(Si_I_) complex or the higher order member of the family. 

In the present study, we have used extensive density functional theory (DFT) calculations to study the structure and energetics of the C_i_C_s_(Si_I_)_n_ defects (where n = 0, 1, 2). Based on these calculations, we propose the structure of the C_i_C_s_(Si_I_)_2_ defect.

## 2. Methodology

### 2.1. Details of Calculations

The calculations in this study were performed using the plane wave DFT code Cambridge Serial Total Energy Package (CASTEP) [29]. To account for the exchange and correlation interactions, we implemented the corrected density functional of Perdew, Burke, and Ernzerhof (PBE) [30], within the generalized gradient approximation (GGA) and with ultrasoft pseudopotentials [31]. A 250-atomic site supercell (5 × 5 × 5 supercell with 2 atoms/primitive cell) was employed under constant pressure conditions. The cut-off of the plane wave basis set was 350 eV, and a 2 × 2 × 2 Monkhorst-Pack (MP) [32] k-point grid was used. This methodology has been employed and discussed in recent studies [33,34,35].

### 2.2. Definitions of Binding Energies

To distinguish between the different configurations of the C_i_C_s_(Si_I_)_n_ defect and to decide which one is favourable, the criterion is the minimization of the binding energy. For example, the binding energy to form a C_i_C_s_(Si_I_)_2_ defect in Si is

E_b_[C_i_C_s_(Si_I_)_2_Si_N_)] = E[C_i_C_s_(Si_I_)_2_Si_N_)] − E[C_i_Si_N_] − E[C_s_Si_N−1_] − 2E[Si_I_Si_N_] − 3E[Si_N_] (1)

where E[C_i_C_s_(Si_I_)_2_Si_N_] is the energy of an N lattice site supercell (here N = 250) containing N Si atoms, a C_i_, a C_s_ atom, two Si_I_‘s, and N Si atoms; E[C_i_Si_N_] is the energy of a supercell containing a C_i_ and N Si atoms; E[C_s_Si_N−1_] is the energy of a supercell containing one C_s_ atom and N − 1 Si atoms; E[Si_I_Si_N_] is the energy of a supercell containing an Si_I_ and N Si atoms; E[Si_N_] is the energy of the N Si atom supercell. A binding energy effectively means that the defect cluster is stable with respect to its constituent point defect components.

## 3. Results and Discussion

Here we have applied extensive DFT calculations to find the lowest energy structures of the C_i_C_s_(Si_I_)_n_ defects (n = 1, 2, …). In essence, we used a step-by-step approach, first calculating the minimum energy structure of C_i_C_s_ and then adding one and finally two Si_I_’s. To explore all possible configurations, we considered numerous calculations (more than 3000) by positioning the constituent defects in all possible sites in the supercell. 

**Figure 1:**
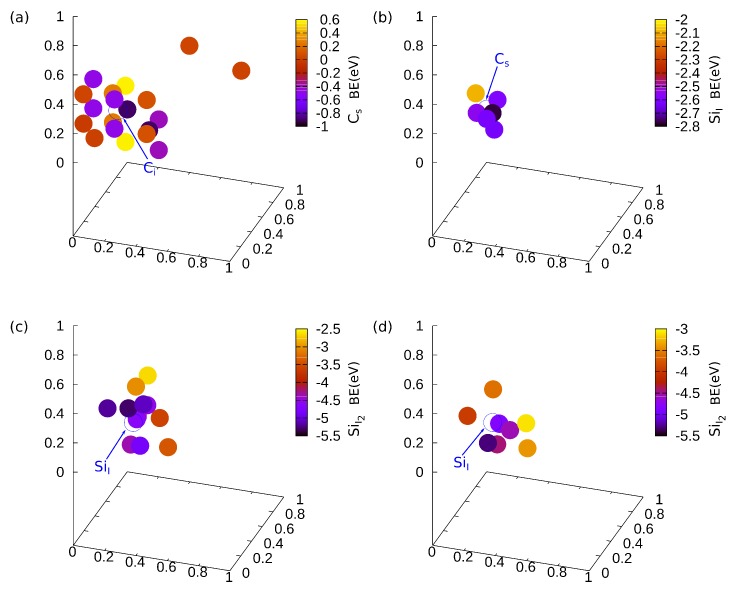
Relative positions of the defects with the lowest binding energy (**a**) of the possible C_s_ substitutional and the C_i_ interstitial defect, (**b**) of the “first in order” possible Si interstitial (Si_I_) and the chosen C_s_ substitutional defects, (**c**) of the “second in order” possible Si interstitial (Si_I2_) and the chosen “first in order” most favourable Si interstitial (Si_I_), and (**d**) of the “second in order” possible Si interstitial (Si_I2_) and the chosen “first in order” second favourable Si interstitial (Si_I_). The blue circle denotes the position of the “given” interstitial and the color bar represents the binding energy for each position.

In the present study, we worked based on the following steps: (a) Knowing the most favourable position for C_i_ [10], we determined the binding energy and the position of the C_s_ substitutional defect for the near and the distant positions in Si positions in the crystal. We found two equivalent positions with a binding energy of −0.90 eV (see Figure 1a). (b) The second step was to add an Si interstitial (Si_I_) to the crystal with the most favourable C_s_ defect. We performed, for this reason, 1000 calculations in a 10 × 10 × 10 grid in a 13.5 Å × 13.5 Å × 13.5 Å cube around C_i_ and C_s_ defects. The binding energy of the most favourable Si_I_ is −2.77 eV (see Figure 1b). (c) The next step was to add a second Si interstitial. Following the same procedure, we performed 1000 calculations in a 10 × 10 × 10 grid in a 13.5 Å × 13.5 Å × 13.5 Å cube in order to determine the position and the binding energy of the “second in order” Si interstitial (Si_I2_) (see Figure 1c). For a better investigation, we also performed the same procedure for the second favourable Si_I_. Thus, we ended up with two equivalent positions with a binding energy of −5.30 eV (see Figure 1d). 

**Figure 2:**
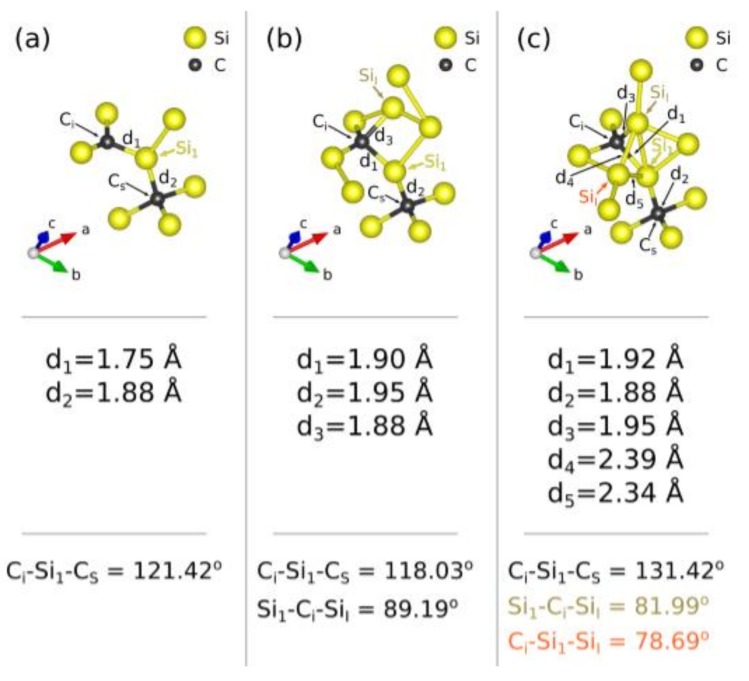
Schematic representation of the energetically favourable (**a**) C_i_C_s_, (**b**) C_i_C_s_(Si_I_), and (**c**) C_i_C_s_(Si_I_)_2_ configurations.

In agreement with previous DFT studies ([36,37] and references therein), the energetically favourable structure of the C_i_C_s_ defect is given in Figure 2a. In this configuration, an Si (marked Si_1_ in what follows) at its lattice site separates the C_i_ and C_s_ atoms. The angle of C_i_Si_I_C_s_ is 121.42° (refer to Figure 2a). The binding energy of the C_i_C_s_ defect is calculated to be −0.90 eV, in agreement with previous studies [21,36,37]. Thereafter, we formed the C_i_C_s_(Si_I_) by adding an Si_I_ to the C_i_C_s_ defect. In this defect, the Si_I_ atom is 1.88 Å away from the C_i_ atom (d_3_ in Figure 2b), whereas the distances between the carbon defects and the bridging Si atom are significantly extended compared to the C_i_C_s_ defect (compare distances d_1_ and d_3_ in Figure 2a,b, respectively). Additionally, the presence of the Si_I_ decreases the angle of C_i_Si_I_C_s_ to 118.03°. This defect is bound with −2.77 eV; i.e., there is an increase in the binding energy by −1.87 eV when an Si_I_ is associated with the C_i_C_s_ defect (refer to Table 1).

The addition of a further Si_I_ results in the formation of the C_i_C_s_(Si_I_)_2_ defect (refer to Figure 2c). The binding energy of this defect is −5.30 eV, so there is an increase of the binding energy by −2.53 eV when an Si_I_ is associated with the C_i_C_s_(Si_I_) defect (refer to Table 1). In this configuration, the first Si_I_ atom is again in the vicinity of the C_i_ atom, but the d_3_ distance increases to 1.95 Å (refer to Figure 2c). The second Si_I_ atom is close to the bridging Si_1_ atom (d_5_ distance being 2.34 Å, Figure 2c). The introduction of the second Si_I_ atom in the vicinity of the Si_1_ atom leads to the extension of the C_i_Si_I_C_s_ angle to 131.42°. The distance between the two Si_I_ atoms is 2.39 Å (d_4_ distance, Figure 2c).

Here, the picture is consistent with the experimental viewpoint that the C_i_C_s_ and C_i_C_s_(Si_I_) defects will attract Si_I_. In essence, when there is a supersaturation of Si_I_, these defects will strongly attract and immobilize the Si_I_. The concentration of the C_i_C_s_(Si_I_)_n_ defects will depend upon the initial carbon concentration of Si and will also require a non-equilibrium concentration of Si_I_ defects. The latter is because of the high formation energy of Si_I_ under equilibrium conditions; therefore, C_i_C_s_(Si_I_)_n_ defects will acquire high concentrations under irradiation or implantation where Si_I_‘s are more abundant. The accumulation of Si_I_ in the C_i_C_s_(Si_I_)_n_ defects will impact the properties of Si and the concentration of unbound Si_I_.

## 4. Conclusions

In the present study, DFT calculations were employed to calculate the binding energies and is in essence the structure of the C_i_C_s_(Si_I_)_n_ defects (n = 0, 1, 2). The structure calculated for C_i_C_s_ is in excellent agreement with previous reports. Here we have calculated the lowest energy structures of the C_i_C_s_(Si_I_) and C_i_C_s_(Si_I_)_2_ defects by employing a comprehensive technique where all possibilities in a 250-atom supercell are considered. It is calculated that the C_i_C_s_(Si_I_) and C_i_C_s_(Si_I_)_2_ defects are strongly bound with −2.77 and −5.30 eV, respectively. This is consistent with experimental work. The electronic structure and energetics of the derived structures should be further investigated using state-of-the-art hybrid functionals. Although the more simple approach used here will yield the right trends, it is anticipated that a hybrid functional will be more appropriate for a detailed understanding of these defects. This study mainly focuses on the binding energies of the C_i_C_s_(Si_I_)_n_ defects, but it should be stressed that kinetics can play a role in the formation of these defects. Atomistic modelling work in conjunction with thermodynamic models (mass action analysis) [38] is needed to assess the relative importance of the C_i_C_s_(Si_I_)_n_ defects as compared to competing carbon-related defects such as C_i_O_i_(Si_I_)_n_.

## Figures and Tables

**Table 1:** The binding energies of the C_i_C_s_(Si_I_)_n_ defects (n = 0, 1, 2) in Si.

Title	BE/eV	BE Difference/eV
C_i_C_s_	−0.90	-
C_i_C_s_(Si_I_)	−2.77	−1.87
C_i_C_s_(Si_I_)_2_	−5.30	−2.53
